# Chirurgie thyroïdienne dans le contexte de populations avec de faibles ressources à l'hôpital dominicain Saint Martin de Porres (Yaoundé, Cameroun)

**DOI:** 10.48327/mtsi.v4i1.2024.443

**Published:** 2024-03-19

**Authors:** Arturo García PAVÍA, Fernando PEREIRA PÉREZ, Iñaki ERQUICIA PERALT, María Isabel HERRERA LÓPEZ, Eva María BURGOS JIMÉNEZ, Akana NGATIA ALEX, Ebune Jackson LOKILI

**Affiliations:** 1 Hôpital dominicain St Martin de Porres, Yaoundé, Cameroun; 2 Hôpital universitaire Puerta de Hierro Majadahonda, Madrid, Espagne; 3 Hôpital universitaire de Fuenlabrada, Madrid, Espagne; 4 Hôpital universitaire Gregorio Marañon, Madrid, Espagne

**Keywords:** Thyroïde, Goitre, Coopération, Thyroïdectomie, Hypocalcémie, Dysphonie, Hôpital dominicain Saint Martin de Porres, Yaoundé, Cameroun, Afrique subsaharienne, Thyroid, Goiter, Cooperation, Thyroidectomy, Hypocalcemia, Dysphonia, Saint Martin de Porres Dominican Hospital, Yaounde, Cameroon, Sub-Saharan Africa

## Abstract

**Introduction:**

Les résultats des campagnes de chirurgie thyroïdienne dans des populations disposant de faibles ressources sont rarement évalués.

**Matériel et méthodes:**

Une campagne de 11 jours a été réalisée à l'hôpital dominicain Saint Martin de Porres. Les données démographiques, les valeurs de TSH, la chirurgie et les complications après 12 mois de suivi ont été analysées.

**Résultats:**

Trente-deux patients ont été opérés. Treize patients avaient un goitre bilatéral, 10 patients un goitre grade II de l'OMS. Dix-huit hémithyroïdectomies (lobo-isthmectomie), 13 thyroïdectomies totales et 1 thyroïdectomie totalisante ont été réalisées. À 12 mois, 3 patients présentaient une dysphonie légère et un patient présentait une hypocalcémie persistante. Le suivi a été effectué directement pour 24 patients ou par téléphone (8 patients) pour ceux ne pouvant se déplacer.

**Conclusions:**

Une série d’étapes importantes doit être respectée : participation active de l'entourage du patient, échographie thyroïdienne par l’équipe chirurgicale pour décider de la technique, sensibilisation intense à la surveillance et à l'hormonothérapie substitutive, et participation du personnel local pour un contrôle à long terme.

## Abstract (en)

**Introduction:**

Surgical campaigns for thyroid surgery in low-income environments are very efficient, but there is little literature reporting results. These campaigns are complex due to multiple particularities: highly evolved cases, the need for professionals to travel or an obvious socio-cultural barrier influence towards the surgical act. We describe a surgical campaign in Cameroon to treat patients with goiter and issue some medical and sociocultural recommendations in view of our experience for its implementation with guarantees.

**Material and methods:**

An experienced group carried out an 11-day campaign at the Saint Martin de Porres Dominican Hospital, Yaounde, Cameroon. Demographic data, TSH values, surgery and complications after a 12-month follow-up were analyzed.

**Results:**

Thirty-eight patients with goiter were selected for the campaign and 32 patients (mean age, 40-years-old; 30 females) were operated. Bilateral goiter, as assessed with echography, was diagnosed in 13 patients (41%). Ten patients (31%) had a WHO grade II goiter (visible with the neck in a normal position). The surgical procedures were 18 unilateral thyroidectomy with isthmectomie, 13 total thyroidectomy, and 1 totalizing thyroidectomy, due to previous unilateral thyroidectomy (cancer recurrence). A pathological study in 13 patients (40%, extra cost 60 €) showed benign multinodular goiter/thyroid nodule (12 patients) and an extensive papillary carcinoma (one patient). Six months postoperatively, 3 patients had a slight dysphonia and one patient had persistent hypocalcemia. Follow-up was completed in all patients, either face to face (75%, 24 patients) or by phone (25%, 8 patients who failed to have a TSH test because of its cost, 23 €).

**Conclusions:**

Surgical campaigns to treat thyroid pathology can be carried out with guarantees if a series of important steps are followed: active participation of the patient's environment, thyroid ultrasound by the surgical team to decide which technique, intense awareness about monitoring and hormone replacement therapy, and the participation of local personnel for long-term follow-up.

## Introduction

La pathologie thyroïdienne au Cameroun a une prévalence élevée due à plusieurs causes, mettant en avant la carence en iode [15, 16] et l'ingestion d'aliments goitrigènes qui bloquent l'absorption et l'utilisation de l'iode [12]. L'accès aux soins de santé au Cameroun, onéreux, devient de moins en moins possible quand on s’éloigne des principales villes. Le pays fournit des efforts importants pour permettre d'accéder à certains traitements comme les antirétroviraux [6] ou les antituberculeux. La santé maternelle et infantile a été améliorée grâce au financement public de certains soins pendant la grossesse et l'accouchement [9]. Cependant, en dehors de ces programmes spécifiques, il n'existe aucun financement pour d'autres traitements. Dans ce contexte, les campagnes chirurgicales menées dans différentes régions du pays représentent la seule opportunité pour de nombreux patients. La pathologie thyroïdienne est cependant difficile à inclure dans ce type de campagnes car certaines de ses complications/séquelles classiques, immédiates (hypocalcémie, lésion du nerf récurrent) ou tardives (hypothyroïdie) peuvent devenir des menaces mortelles dans des environnements aux ressources limitées.

L'objectif de ce travail est de décrire les résultats d'une série de thyroïdectomies et d'en montrer les particularités.

## Matériel et méthodes

Nous présentons les résultats obtenus après intervention sur 32 patients atteints d'une pathologie thyroïdienne à l'hôpital dominicain Saint Martin de Porres à Yaoundé (capitale du Cameroun) pendant 11 jours en novembre 2021. L’équipe chirurgicale était composée de 2 anesthésistes, 2 chirurgiens généralistes avec expérience en chirurgie thyroïdienne et une infirmière instrumentiste, associée à du personnel hospitalier local : infirmières de bloc opératoire, infirmières anesthésistes et médecins. L'un des chirurgiens espagnols travaillait en permanence dans le centre camerounais, ce qui était essentiel pour la sélection des patients et pour leur suivi ultérieur. En accord avec la direction de l'hôpital, un prix total de 175 000 FCFA (270 fi) par patient a été demandé. Il s'agissait d'un prix à but non lucratif qui comprenait l'ensemble du processus hospitalier ainsi que les visites de suivi postopératoires.

Le comité d’éthique de l'hôpital a approuvé l’étude et validé la collecte des données des patients.

Tous les patients ont eu un examen sanguin préopératoire général (hémogramme, coagulation et sérologie VIH) ainsi qu'un dosage de la TSH (Thyroid Stimulating Hormone). Aucune calcémie préopératoire n’était disponible. Le processus de sélection des patients a suivi un circuit standard de consultation, demande de tests préopératoires, étude hormonale, interprétation des résultats, proposition d'intervention et signature du consentement éclairé. Au cours des deux mois précédant la campagne, 307 patients ont été examinés avec une sélection finale de 32 ayant complété le bilan préopératoire. Tous les patients avaient du sang réservé avant l'intervention.

L'information concernant la chirurgie, les complications et le suivi nécessaire a été réalisée en présence d'une personne responsable de la famille, avec un accent particulier sur la nécessité éventuelle d'un traitement hormonal ou calcique à vie.

Les techniques prévues étaient une hémithyroïdectomie avec isthmectomie en cas de pathologie unilatérale évaluée par échographie et une thyroïdectomie totale en cas de pathologie bilatérale évaluée par échographie. Une calcémie était réalisée 24 heures après l'intervention en cas de thyroïdectomie totale. Les patients subissant une hémithyroïdectomie sortaient sans traitement hormonal. Ceux avec une thyroïdectomie totale sortaient avec de la lévothyroxine orale à la dose de 1,5 µg/kg/ jour, ce traitement étant assuré par l'hôpital. Des instructions précises étaient données pour se rendre à un contrôle post-opératoire à 4 semaines. Lors de cette consultation, les taux de TSH étaient demandés à 6 semaines afin d’établir le traitement hormonal. Un nouvel examen était programmé à 6 mois et à 12 mois. Il n'a pas été possible de réaliser des laryngoscopies pour évaluer les cordes vocales. L’étude anatomopathologique de la pièce n'a pu être réalisée que chez des patients assumant le surcoût (40 000 FCFA soit 60 €).

## Résultats

**Figure 1:**
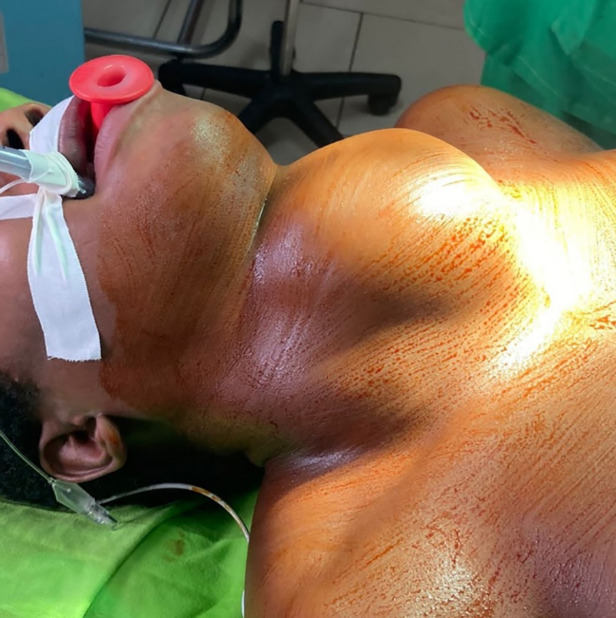
Jeune femme atteinte d'un goitre de grade III Young woman with grade III goiter

Trente femmes et 2 hommes ont été opérés, avec un âge moyen de 40 ans. Parmi eux, 26 étaient des cas avancés (WHO goitre grade II ou grade III). Les données démographiques et préopératoires sont présentées dans le tableau I. Quinze opérés avaient une voix normale le lendemain, 12 une dysphonie légère. Cinq thyroïdectomies totales présentaient un stridor traité par corticoïdes intraveineux et inhalés, avec une bonne évolution. Parmi ces 5 cas de stridor, 3 étaient des goitres de grande taille (grade III de l'OMS, visibles à distance) (Fig. 1), 1 était un goitre associé à une maladie de Basedow non contrôlée par un traitement depuis 2 ans et 1 cas était une thyroïdectomie totalisante due à une masse cervicale néoplasique avec résection de la veine jugulaire infiltrée et ayant subi une hémithyroïdectomie dans le passé. Les complications chirurgicales sont reflétées dans le tableau II. Chez les 14 patients ayant subi une thyroïdectomie totale (y compris une hémithyroïdectomie totalisante), une calcémie totale a été mesurée 24 heures après la chirurgie : 12 présentaient une hypocalcémie, sévère seulement chez un patient, 6 mg/dl, les autres légères (calcémie ≥ 7,5 mg/dl).

**Tableau I:** Données démographiques et préopératoires Demographic and preoperative data

**Données démographiques**	
hommes	2 cas
femmes	30 cas
âge	40 (18-66)ans
**Classification goitre (n=32)**	
goitre grade IB	6 cas
goitre grade II	16 cas
goitre grade III	10 cas
**Valeurs préopératoires de TSH (N = 0,27-4,2)**	
TSH normal	25 cas
TSH <0,01	1 cas
TSH légèrement bas (0,1-0,27)	4 cas
TSH élevé (>4,2)	2 cas
**Intervention chirurgicale**	
unilatérale (HT)	18 cas
bilatérale (TT)	13 cas
thyroïdectomie totalisante	1 cas

HT : Hémithyroïdectomie TT : Thyroïdectomie totale

**Tableau II:** Complications chirurgicales Surgical complications

	Événements
Saignement (réintervention)	3
Séromes superficiels	7
Infection de la plaie	1
Chéloïde	1
Hypocalcémie postopératoire en TT* (n=14)	12
Dysphonie précoce	12
Stridor	5
Dysphonie 6 mois	3

TT : Thyroïdectomie totale

* calcémie postopératoire réalisée uniquement en TT

Le patient en hypocalcémie sévère était obèse morbide, avec un goitre de grade III, et il présentait un stridor. Après deux jours de traitement par calcium intraveineux, ses symptômes se sont améliorés et le taux de calcium est passé à 7,8 mg/dl, ce qui lui a permis de sortir avec un traitement oral.

L’étude anatomo-pathologique a pu être réalisée sur 13 patients : 12 cas de goitre multinodulaire/nodule thyroïdien bénin et 1 cas de carcinome papillaire étendu, déjà suspecté lors de l'intervention chirurgicale. Tous les patients se sont présentés au rendez-vous à 4 semaines mais seulement 24 ont eu un dosage de TSH à 6 semaines et sont revenus avec les résultats. Les 8 patients n'ayant pas eu de dosage de TSH postopératoire (3 hémithyroïdectomies et 5 thyroïdectomies totales) ont été contactés par téléphone. Ils ont expliqué que la distance, le coût des analyses (environ 15 000 FCFA) et surtout le fait de se sentir bien, étaient la raison de leur interruption du suivi. Lors des visites de 6 mois et de 12 mois, les mêmes patients que ceux de la visite à 6 semaines étaient présents. Les 8 autres ont été recontactés par téléphone ce qui a permis de s'assurer que les 3 hémithyroïdectomies ne présentaient pas de symptômes évidents d'hypothyroïdie et que les 5 thyroïdectomies continuaient à prendre le traitement hormonal. Pour les 14 thyroïdectomies totales, la dose a dû être ajustée chez 4 des 9 patients ayant eu un dosage de TSH (valeur normale 0,4-4,0 mUI/l) à 6 semaines et 6 mois. Deux goitres multinodulaires avaient une TSH élevée (12 et 5,34 mUI/l) et le patient atteint de la maladie de Basedow avait au 6^e^ mois une TSH très élevée (35 mUI/l) mais sans symptômes. Dans le cas de la thyroïdectomie totalisante pour cancer, les taux de TSH étaient dans les limites normales mais la dose de substitution a été augmentée pour maintenir l'inhibition hormonale. Concernant les 18 hémithyroïdectomies, seule un des 15 patients ayant eu un contrôle de TSH présentait un résultat élevé (9 mUI/l) à 6 semaines et un traitement substitutif a été prescrit. A 6 mois, les 12 dysphonies légères avaient une récupération complète de la voix. Parmi les 5 patients ayant présenté un stridor, 2 avaient une voix normale et 3 une légère dysphonie. Ainsi, à 6 mois, 29 des 32 patients avaient une voix normale.

## Discussion

La pathologie thyroïdienne, dans un contexte comme celui du Cameroun, évolue généralement de nombreuses années avant d’être vue en consultation [12]. Son traitement nécessite un investissement économique important : consultation d'un spécialiste, étude hormonale et si une intervention chirurgicale est nécessaire, elle n'est réalisée que dans des centres dotés de rares spécialistes qualifiés. Le prix normal d'une thyroïdectomie peut varier entre 600 000 et 1 000 000 FCFA (1 000 à 1 500 €). Dans un pays où le revenu mensuel moyen tourne autour de 100 000 à 130 000 FCFA), cette somme est difficile à trouver pour le patient et sa famille.

Les conditions avantageuses de la campagne promue par l'hôpital St Martin de Porres ont permis de prendre en charge des patients venus de différentes régions du pays.

### Technique chirurgicale

Dans une population avec de faibles revenus, il est classiquement recommandé, en cas de maladie bilatérale, de recourir à une thyroïdectomie subtotale d'au moins un des côtés pour tenter de prévenir les complications liées à la lésion des nerfs laryngés ainsi que celles d'une parathyroïdie bilatérale et pour préserver un certain degré de fonction thyroïdienne. Il s'agit d'une technique qui n'est pas recommandée dans les pays plus développés en raison du risque de récidive du goitre [17]. Ce risque est plus grand si le fragment laissé est nodulaire (comme c'est généralement le cas dans les gros goitres) et encore plus s'il n'y a pas de possibilité de substitution hormonal. La prévention de l'hypothyroïdie par des thyroïdectomies subtotales bilatérales (TSB) est illusoire, comme nous l'avons observé dans un précédent travail [10]. Dans le cas des hémithyroïdectomies, on pourrait penser que cet argument n'est pas important, compte tenu du faible taux d'hypothyroïdie postchirurgicale. Cependant, plusieurs études ont montré une incidence d'hypothyroïdie post-chirurgicale après hémithyroïdectomie allant jusqu’à 27 ***%*** [8, 14], éventuellement plus élevée dans un environnement comme celui du Cameroun [10]. L'analyse de la TSH doit également être réalisée après 6 semaines, avec un suivi annuel. Notre recommandation est de ne réaliser aucun type de thyroïdectomie bilatérale s'il n'existe aucune possibilité d’évaluer la fonction thyroïdienne ultérieure et de la traiter. Autrement dit, il est indispensable assurer le contrôle hormonal en période postopératoire si une thyroïdectomie bilatérale est indiquée. Il semble raisonnable de réaliser des hémithyroïdectomies lorsqu'il n'existe aucune possibilité de traitement de substitution. De nombreux cas sont unilatéraux (56 % dans notre échantillon). Cependant, la fiabilité de l'examen clinique pour déterminer si la maladie est unilatérale ou bilatérale est faible, notamment des gros goitres. Seule une échographie détermine en toute sécurité la latéralité du goitre. Par conséquent, une deuxième recommandation concerne l'utilisation généralisée de l’échographie cervicale pour la sélection des patients [2]. Compte tenu de sa large disponibilité et de la facilité d'utilisation des équipements portables, cela ne devrait pas constituer un inconvénient majeur.

Nous avons choisi de ne pas réaliser de TSB parce que nous avions deux chirurgiens qualifiés pour l'identification des nerfs récurrents et des parathyroïdes, nous étions en milieu hospitalier et nous étions assurés de l'approvisionnement en calcium et hormones. Si ce n'est pas le cas, comme cela arrive en milieu rural, en cas de maladie bilatérale il est logique de laisser des restes thyroïdiens sur au moins un des côtés afin de minimiser le risque de lésion du nerf laryngé et d'hypoparathyroïdie tout en sachant que cette attitude n'empêche pas l'hypothyroïdie [10].

### Traitement hormonal postopératoire

Le remplacement hormonal est indispensable, particulièrement important chez les patients présentant des taux élevés de TSH, chez les femmes en âge de procréer (en raison des conséquences graves de l'hypothyroïdie lors d'une éventuelle grossesse), si le reste de la thyroïde est volumineux (pour éviter la récidive du goitre due à une stimulation continue) et lorsqu'il y a un diagnostic de cancer. Le prix du traitement hormonal substitutif n'est pas élevé. Au Cameroun, une boîte de 100 comprimés de lévothyroxine coûte environ 3 200 FCFA (5 €). Le principal problème est la prise de conscience par les patients de la nécessité de surveiller, d'analyser et de suivre un traitement quotidiennement et à vie. Cette circonstance, combinée au fait que la lévothyroxine orale est difficile à obtenir en dehors des grandes villes, rend l'observance thérapeutique difficile, en particulier chez les patients qui vivent en zone rurale. Il s'agit d'un risque grave pour la santé des patients subissant une thyroïdectomie bilatérale. À ce stade, l'implication de la famille est cruciale pour la réussite du processus à long terme. La signature du consentement éclairé par un représentant de la famille, après dialogue avec le chirurgien, facilite le suivi ultérieur. Face à une intervention chirurgicale, il y a souvent une participation étroite des responsables de la famille. Leur rôle doit être reconnu. Notre recommandation est de bien sensibiliser au traitement postopératoire et d’éviter la chirurgie bilatérale s'il n'y a pas d'engagement de la part du patient et de sa famille à se soumettre à des études hormonales périodiques, ou s'ils n'ont pas la possibilité d'un apport chronique d'hormones thyroïdiennes. Cet aspect ne devrait pas être un inconvénient majeur lors de la planification de ce type de campagne. Les associations peuvent faciliter l'accès au traitement initial, mais généralement pas à long terme. Dans notre travail, les 15 patientes sous traitement hormonal (14 TT et 1 HT) ont continué à en acheter et à le prendre un an après l'intervention.

### Suivi postopératoire

Dans ce type de campagnes chirurgicales, le suivi est très variable, allant de 30 % à 67 % [4, 7]. Même en considérant dans notre campagne qu'un des chirurgiens vivait au Cameroun, qu'il avait sensibilisé les patients et leurs familles à la possibilité et à la gravité de l'hypothyroïdie post-chirurgicale, et qu'il était vigilant pour éviter des pertes de vue, jusqu’à un quart des patients n'ont pas fait l’étude hormonale convenue et n'ont pu être évalués que par téléphone. Le traitement substitutif a dû être ajusté fréquemment (7 % des HT et 28 % des TT). Nous soulignons donc que, dans le cas des thyroïdectomies bilatérales, le suivi est essentiel, idéalement en disposant de personnel local pour le faire.

### Hypocalcémie postopératoire

Initialement, la décision de commencer un traitement par calcium oral après une thyroïdectomie, devait être guidée par les données de calcémie postopératoire. Mais quand il a été confirmé que la majorité des patients présentait une hypocalcémie complètement asymptomatique, nous avons décidé d’être guidés par la clinique. Les patients présentant une hypocalcémie modérée postopératoire (supérieure à 7,5 mg/dl) sans symptômes associés, n'ont pas reçu de traitement. L'absence de données sur la calcémie préopératoire, sur le calcium ionique ou l'albumine, rend impossible le recours aux recommandations en vigueur dans les pays plus développés. Les 2 cas présentant des symptômes légers ont bien évolué et leur traitement a été interrompu après un mois. Le seul cas qui présentait initialement des taux de calcium très faibles a continué à avoir besoin de calcium par voie orale 6 et 12 mois après l'intervention, ce qui fait suspecter une hypoparathyroïdie post-chirurgicale persistante. Nous recommandons d’évaluer les taux de calcium total postopératoires avec prudence et de se guider par la clinique lors de la prescription du traitement [4, 7].

### Dysphonie post-opératoire

La possibilité d’évaluer la fonction des cordes vocales ajoute un point incontestable de sécurité en chirurgie thyroïdienne [13]. La disponibilité d’équipements de laryngoscopie portables, peu coûteux et faciles à utiliser, nous permet aujourd'hui de faire cette évaluation. Notre groupe ne disposait pas de cet équipement pour ses travaux, mais a pu l'utiliser lors de campagnes ultérieures. Nous considérons qu'ils constituent un outil utile dans l’évaluation préopératoire et qu'ils doivent être utilisés en postopératoire pour évaluer correctement la dysphonie.

### Hématomes/séromes post-opératoires

Les 3 cas d'hématomes sont à noter. Ils s'expliquent par la défaillance du dispositif utilisé, un modèle obsolète dans lequel nous avions placé une confiance excessive. Après avoir eu recours aux ligatures classiques, nous n'avons plus eu de problème. Dans tous les cas, une extrême vigilance doit être exercée au réveil des patients et il convient d'anticiper les besoins en transfusion sanguine. L'utilisation de drains en chirurgie thyroïdienne est un sujet en constante évolution. La tendance est de ne pas les utiliser [11] et la littérature le soutient dans de nombreux articles [5]. Cependant, lors des campagnes chirurgicales, notre équipe les utilise systématiquement en raison de la taille des goitres, en accord avec ce qui est fait par d'autres équipes [7].

### Chéloïdes

L'apparition de chéloïdes chez les patients à peau noire est fréquente [3]. Dans le cas de cicatrices visibles, comme les cicatrices cervicales, il est important que les patients puissent se voir proposer un éventuel traitement. Un patient présentait une chéloïde de taille importante à 6 mois. Une infiltration sous-cutanée de triamcinolone 20 mg/ml (1 ml) a été réalisée, suivie d'une seconde infiltration après 4 semaines, avec quasi-disparition d'une chéloïde. C'est un corticostéroïde facilement accessible et qui a montré de bons résultats [1].

## Conclusions

Au vu de notre expérience, le traitement des pathologies thyroïdiennes doit tenir compte des particularités de la population à traiter, en s'appuyant toujours sur les structures et professionnels de santé locaux. Ces structures doivent assurer une préparation correcte des patients et un suivi exhaustif après l'intervention. L'incidence des complications peut être minimisée en concentrant, de manière ordonnée, plusieurs actions réalisées par une équipe expérimentée. Le principal obstacle pour les patients est le coût économique. Comme limites de notre travail, nous devons considérer que ces résultats ne peuvent pas être extrapolés à d'autres contextes. Il peut encourager d'autres professionnels de la santé et les institutions à promouvoir des actions similaires.

## Remerciements

Nous remercions tout le personnel camerounais de l'hôpital dominicain St Martin de Porres pour son dévouement quotidien afin d'offrir de meilleures conditions de santé à leurs concitoyens. Nous remercions l'ONG Zerca y Lejos pour son soutien ainsi que l'hôpital universitaire Puerta de Hierro, l'hôpital universitaire de Fuenlabrada et l'hôpital universitaire Gregorio Maranon pour le matériel chirurgical fourni.

## Sources de financement

Ce travail n'a reçu aucun financement d'aucune source.

## Contribution des auteurs

García Pavía A : conception de l’étude, rédaction, révision et validation du protocole, recueil des données, analyse, rédaction et correction du manuscrit.

Pereira Pérez F : conception de l’étude, rédaction, révision et validation du protocole, recueil des données, analyse, rédaction et correction du manuscrit.

Erquicia Peralt I : conception de l’étude, révision et validation du protocole, recueil des données, correction du manuscrit.

Herrera López MI : conception de l’étude, révision et validation du protocole, recueil des données, correction du manuscrit.

Burgos Jiménez EM : conception de l’étude, révision et validation du protocole, recueil des données, correction du manuscrit.

Ngatia Alex A : conception de l’étude, révision et validation du protocole, recueil des données, correction du manuscrit.

Lokili EJ : conception de l’étude, révision et validation du protocole, recueil des données, correction du manuscrit.

## Conflit d'intérêts

Les auteurs déclarent qu'ils n'ont aucun conflit d'intérêts.
